# Construction and Validation of Novel Ferroptosis-related Risk Score Signature and Prognostic Prediction Nomogram for Patients with Colorectal Cancer

**DOI:** 10.7150/ijms.91446

**Published:** 2024-04-22

**Authors:** Ruibin Liu, Yue Wang, Jiawen Bu, Qingqing Li, Fang Chen, Mengying Zhu, Huanyu Chi, Guilin Yu, Tong Zhu, Xudong Zhu, Guohua Zhao

**Affiliations:** 1 Department of General Surgery, Cancer Hospital of Dalian University of Technology, Cancer Hospital of China Medical University, Liaoning Cancer Hospital & Institute, Shenyang, Liaoning 110042, China.; 2 Liaoning University of Traditional Chinese Medicine, Shenyang, Liaoning 110847, China.; 3 Department of Colorectal Surgery, Shengjing Hospital of China Medical University, Shenyang, Liaoning 110004, China.; 4 Department of Endoscopy, Cancer Hospital of Dalian University of Technology, Cancer Hospital of China Medical University, Liaoning Cancer Hospital & Institute, Shenyang, Liaoning 110042, China.; 5 Department of Gynecology, People's Hospital of Liaoning Province, Shenyang, Liaoning 110016, China.; 6 Department of Breast Surgery, Panjin Central Hospital, Panjin, Liaoning 124010, China.; 7 Liaoning Provincial Key Laboratory of Precision Medicine for Malignant Tumors, Shenyang, Liaoning 110042, China.; 8 Markey Cancer Center, University of Kentucky, Lexington, KY 40536, United States of America.

**Keywords:** Colorectal Cancer, Ferroptosis, Risk model, Nomogram, Prognosis

## Abstract

**Background:** Colorectal cancer (CRC) has a high morbidity and mortality. Ferroptosis is a phenomenon in which metabolism and cell death are closely related. The role of ferroptosis-related genes in the progression of CRC is still not clear. Therefore, we screened and validated the ferroptosis-related genes which could determine the prevalence, risk and prognosis of patients with CRC.

**Methods:** We firstly screened differentially expressed ferroptosis-related genes by The Cancer Genome Atlas (TCGA) database. Then, these genes were used to construct a risk-score model using the least absolute shrinkage and selection operator (LASSO) regression algorithm. The function and prognosis of the ferroptosis-related genes were confirmed using multi-omics analysis. The gene expression results were validated using publicly available databases and qPCR. We also used publicly available data and ferroptosis-related genes to construct a prognostic prediction nomogram.

**Results:** A total of 24 differential expressed genes associated with ferroptosis were screened in this study. A three-gene risk score model was then established based on these 24 genes and GPX3, CDKN2A and SLC7A11 were selected. The significant prognostic value of this novel three-gene signature was also assessed. Furthermore, we conducted RT-qPCR analysis on cell lines and tissues, and validated the high expression of CDKN2A, GPX3 and low expression of SLC7A11 in CRC cells. The observed mRNA expression of GPX3, CDKN2A and SLC7A11 was consistent with the predicted outcomes. Besides, eight variables including selected ferroptosis related genes were included to establish the prognostic prediction nomogram for patients with CRC. The calibration plots showed favorable consistency between the prediction of the nomogram and actual observations. Also, the time-dependent AUC (>0.7) indicated satisfactory discriminative ability of the nomogram.

**Conclusions:** The present study constructed and validated a novel ferroptosis-related three-gene risk score signature and a prognostic prediction nomogram for patients with CRC. Also, we screened and validated the ferroptosis-related genes GPX3, CDKN2A, and SLC7A11 which could serve as novel biomarkers for patients with CRC.

## Introduction

In 2020, colorectal cancer (CRC) was ranked third in incidence and second in mortality among the most prevalent cancers 1. The incidence of CRC is on the rise in countries with an increasing human development index 2. Despite improvements in diagnosis and treatment, some patients with CRC may experience disease progression, leading to less effective therapy and poorer survival outcomes 3-5. Therefore, there is an urgent need to identify the novel prognostic indicators and explore the responding regulatory mechanisms for patients with CRC.

Iron has significant biological importance in the development of CRC and other solid cancer 6, 7. Unlike normal cells, many kinds of tumor cells exhibit a heightened dependence on iron for accelerated growth, rendering them vulnerable to iron depletion. Besides, tumor cells also have a high proliferation rate owing to their addiction to iron 8-10. Ferroptosis is a novel form of cell death represented by dysregulation of iron and related lipid metabolism 11, 12. Dysregulation of ferroptosis has been shown to contribute to the proliferation, invasion, and metastasis of solid cancer cells, including CRC 13-15. As for the specific relationships between ferroptosis and CRC, and the importance of ferroptosis in the development of CRC, it may mainly lie on the function of ferroptosis-related genes. Ferroptosis-related genes significantly regulate molecular changes and physiological activities of CRC cells 16, 17. Abnormal expression of ferroptosis-related genes may affect the occurrence, development and even the treatment sensitivity of CRC. It has been shown that decreased expression of ferroptosis-related gene Glutathione Peroxidase 4 (GPX4) may increase CRC cells' sensitivity to oxidative stress and decreased CRC cells' tolerance to lipid peroxides, which may finally lead to the death of CRC cells 18. Meanwhile, it has also been found that the down-regulation of ferroptosis-related gene Metallothionein-1G resulted in a significant inhibition of CRC cells' proliferation 19. Other studies even found that the enhanced expression and activity of ferroptosis-related genes in CRC cells were also crucial for many kinds of anti-tumor drugs to activate iron-dependent cell death and inhibit CRC development. For example, the use of ferroptosis inducers can significantly reverse Oxaliplatin resistance in CRC cells 20. All these above findings proved that ferroptosis may significantly affect the malignant development of CRC. Ferroptosis-related genes may serve as novel biomarkers and therapeutic targets for the patients with CRC.

Our study aimed to investigate and validate ferroptosis-related genes which could serve as novel prognostic biomarkers for patients with CRC by conducting a comprehensive bioinformatics analysis on The Cancer Genome Atlas (TCGA) datasets, integrating gene expression levels, nomograms, immune infiltration, spatial and single-cell location, DNA methylation, clinical data and Quantitative RT-PCR (RT-qPCR) analysis. In addition, gene set enrichment analysis (GSEA) and gene set variation analysis (GSVA) were also used to explore the potential pathways and mechanisms associated with selected ferroptosis-related genes in the various groups. In conclusion, we screened and validated the differentiated expressed ferroptosis-related genes *GPX3*, *CDKN2A*, and *SLC7A11*, which may serve as novel prognostic biomarkers for patients with CRC. Furthermore, this novel three-gene risk score signature and nomogram could be helpful for monitoring the survival outcomes of patients with CRC.

## Materials and methods

### Assembling ferroptosis-related gene set

Ferroptosis-related genes were retrieved using MSigDB version 7.1 21, 22. This study incorporated previously reported genes identified through literature review 23, 24. A gene set containing 404 ferroptosis-related genes was obtained by eliminating overlapping genes.

### Data sets and processing

Gene expression data for 647 CRC samples from TCGA and 51 normal samples from the GTEx project were downloaded from the UCSC Xena. The dataset included samples from TCGA, TARGET, and GTEx. The R package “TCGA biolinks” was used to retrieve clinical data pertaining to patients with CRC. Gene expression data were collected from patients. Patients with prognostic information-deficient data were excluded from the analysis. Finally, 619 patients were included in TCGA dataset. Ethics committee approval was not required because all data were publicly accessible.

### Differential analysis

Initially, we screened 1765 differentially expressed genes (DEGs) identified in TCGA gene expression matrix. The “DESeq2” package in R software (v 3.6.3) was used to analyze and identify DEGs between TCGA-CRC samples with normal tissues 25-28. DEGs were filtered using an adjusted *P*-value threshold of < 0.05 and an absolute log2-fold change >1. Twenty-four ferroptosis-related genes were selected for downstream analysis. Metascape (https://metascape.org/gp/index.html#/main/step1) was used for functional enrichment analysis of the selected DEGs 29.

### Constructing and validating the risk-score signature

We used Cox proportional hazards regression and least absolute shrinkage and selection operator (LASSO) regression analysis to select 24 differentially expressed ferroptosis-related genes from the TCGA dataset. LASSO regression analysis with 10-fold cross-validation was used for establishing an ferroptosis-related genes-based scoring model to predict the patient's prognosis. The risk score was calculated as follows:

Risk Score = β1 × exp1 + β2 × exp2 + … + βi × exp i

where β = the coefficient of ferroptosis-related genes, and exp = the expression levels of ferroptosis-related genes.

Normalized gene expression levels were calculated using the “edgeR” package. We also calculated a risk score for each available patient with CRC and generated distribution and receiver operating characteristic (ROC) curves using “time ROC” software 30. The patients were stratified into high- and low-risk categories based on their median risk scores. Kaplan-Meier (KM) survival analysis was used to determine the performance of the ferroptosis-related genes-based scoring model in predicting prognosis. The difference in overall survival (OS) was compared in patients with CRC in the two subgroups.

### Functional enrichment analysis

FDR correction was applied to the significant enrichment *P*-values. Pre-ranked GSEA was used to confirm the biological functions and pathways of each module. The Molecular Signature Database v5.1 predefined gene sets were used. The enrichment score was calculated based on the rank order of genes determined by the random forest accuracy. GSVA was conducted using the GSVA package in R software.

### Cell lines and cell culture

The human CRC cell lines SW480, SW620, and HCT116 and the human colonic epithelial cell line NCM460 were procured from the Cell Bank of the Chinese Academy of Sciences (Shanghai, China). The cells were cultured in L-15 or RPMI-1640 medium (Gibco, Carlsbad, USA) supplemented with 10% fetal bovine serum (FBS, Gibco) and 1% penicillin-streptomycin (HyClone, Logan, USA) at 37°C in 5% CO_2_.

### RT-qPCR analysis

RT-qPCR was performed to assess the expression level of GPX3, CDKN2A and SLC7A11 mRNA in CRC cell lines and tissue samples. Total RNA was isolated from the cells or tissue samples using TRIzol reagent (Invitrogen). Complementary DNA (cDNA) was synthesized using the PrimeScript RT Reagent Kit (TaKaRa, Japan). RT-qPCR was performed on an ABI 7500 H T system using SYBR Green PCR master mix (Applied Biosystems, CA, USA) and primers. The specific mRNA expression levels were quantified using the 2^-ΔΔCT^ method. β-actin served as the control for normalization. The primer sequences for RT-qPCR were shown in **Table 1**.

### Analysis of spatial transcriptomics technology

The code for transcriptomic, spatial, and statistical analyses is available in GitHub (https://github.com/lrb0533/CRC-Spatial). RNA-seq data for single-cell studies were collected from public databases (www.10xgenomics.com).

### Analysis of single-cell sequencing data

The Colon Adenocarcinoma (COAD) single-cell dataset, Genomic Spatial Event (GSE), comprising GSM6061704-11773, GSM6061704-15564, and GSM6061704-8810, was obtained from the Gene Expression Omnibus (GEO) database. The dataset comprised 11 samples. Quality control was subsequently performed on the data. We selected cells with <10% mitochondrial genes, >200 total genes, and genes with expression levels between 200 and 6000 expressed in at least three cells. The number of highly variable genes was set to 2000. SCT correction was applied to these 11 samples for integration. The tSNE method was used to reduce data dimensionality by setting the “DIMS” parameter to 20. Cell clustering was performed using the “KNN” method with a resolution of 0.6. These cells were subsequently annotated using various cell surface markers. Finally, the percentage of ferroptosis-related genes in each cell was determined by using the “Percentage Feature Set” function to import the relevant genes.

### Developing and evaluating the nomogram

Univariate and multivariate Cox regression analyses were conducted to analyze the clinicopathological parameters, including T stage, N stage, M stage, age, CEA level, CDKN2A expression, GPX3 expression, and SLC7A11 expression, to determine the independent predictive ability of the risk score system. The clinical characteristics of these patients with CRC were obtained from TCGA-COAD dataset (**Table 2**). The rms package was used to integrate independent prognostic factors and construct a nomogram for predicting the 1-, 3-, and 5-year OS. ROC analysis and calibration were used to evaluate the discriminative ability of the nomogram 31.

### Analysis of the TIMER database and DNA methylation data

Tumor IMmune Estimation Resource (TIMER) enables automated visualization and analysis of immune cell infiltration in all TCGA tumors 32, 33. The TIMER algorithm uses six discrete immune cell subsets to estimate infiltration. The cellular composition includes B cells, CD4^+^ T cells, CD8^+^ T cells, macrophages, neutrophils, and dendritic cells. This study aimed to analyze the results of infiltration estimation and compare immune cell subsets in low- and high-risk groups (http://timer.cistrome.org/) 34. The MethSurv database was used to analyze the DNA methylation sites of ferroptosis-related genes in TCGA database (https://biit.cs.ut.ee/methsurv/).

### Constructing transcription factors (TFs) -mRNA-miRNA networks

The miRNAs and TFs of the three selected ferroptosis-related genes were predicted using TargetScan Human 7.2 and GeneCards. Results were visualized using the Cytoscape software (v3.8.0).

### Statistical analysis

Statistical analyses were performed using GraphPad Prism and the R software. Kaplan-Meier survival analyses were performed using log-rank tests. When applicable, hazard ratios (HRs) and 95% confidence intervals (CIs) were reported. Student's t-test and independent sample t-test were used for two-group comparisons. A two-tailed *P* value of <0.05 was considered statistically significant without explicit annotation.

### Ethical approval and consent to participate

This study was approved by the Ethics Committee of Cancer Hospital of China Medical University. Written informed consent was obtained from all participants, including healthy volunteers and patients who provided clinical specimens.

## Results

### Identification of DEGs associated with ferroptosis in patients with CRC

**Figure 1:**
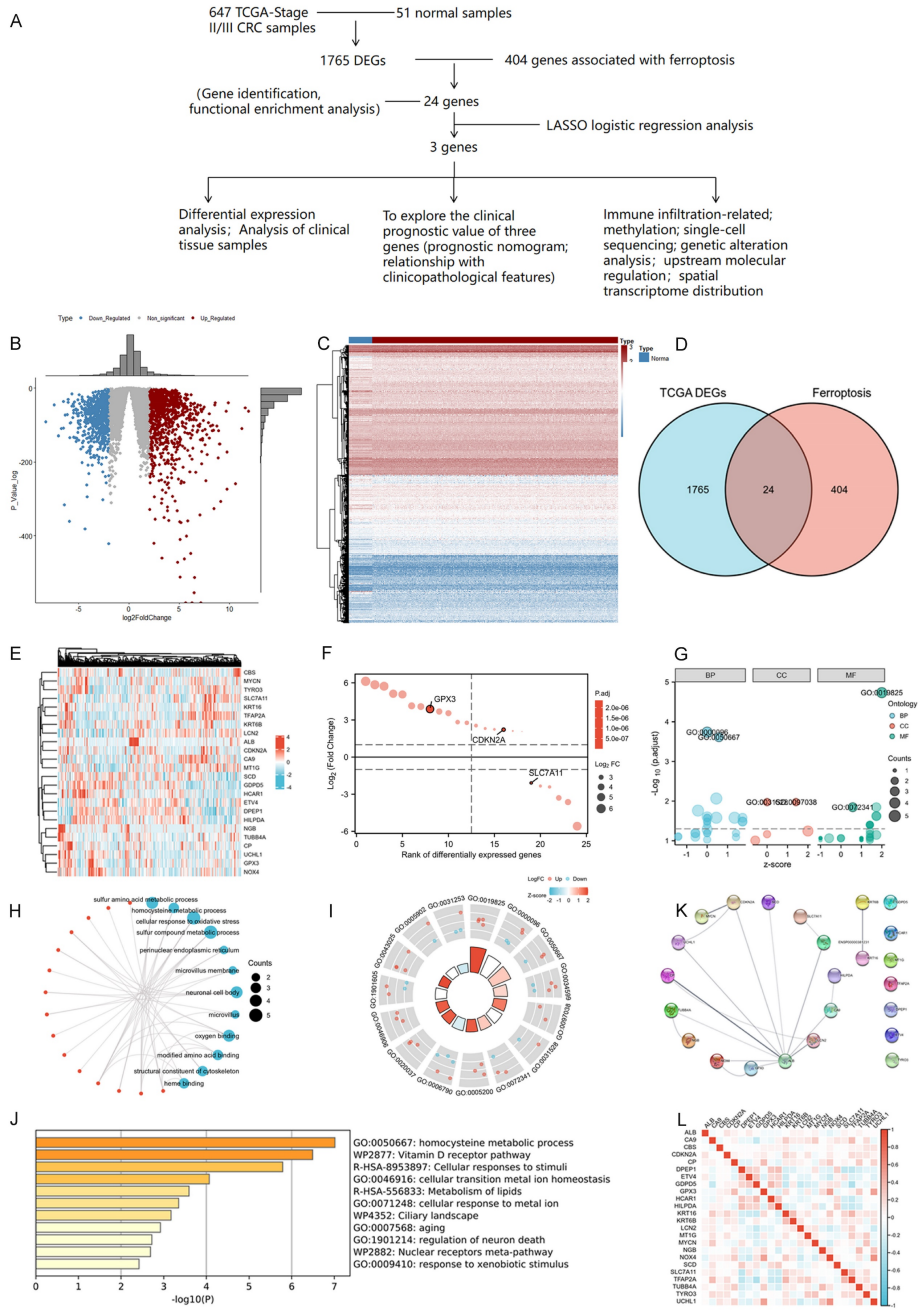
Identification and functional enrichment analysis of dysregulated ferroptosis-related genes in CRC. A: Schematic overview of the whole study. B-C: Differential gene volcano, heatmap of CRC based on TGCA data. D: Venn diagram representing intersections of CRC DEGs and ferroptosis-related genes. E: Heatmap of the expression levels of 24 ferroptosis-related DEGs based on TCGA data. F: Map of ranking of differences of 24 ferroptosis-related DEGs. G-I: Enriched Gene Ontology terms and KEGG pathways of 24 ferroptosis-related DEGs. J: Enriched Gene Ontology terms and KEGG pathways of 24 ferroptosis-related DEGs according to the Metascape. K: The correlation network of 24 ferroptosis-related DEGs according to the STRING database. L: The correlations heatmap of 24 ferroptosis-related DEGs.

We firstly conducted an integrated analysis to investigate ferroptosis-related DEGs in CRC, the schematic overview of the whole study was shown in Figure 1A. A comprehensive analysis of 404 ferroptosis-related genes was conducted using the GeneCard database and literature review. We identified 1765 DEGs in 647 tumor and 51 normal samples from the TCGA COAD database. Volcano and heat maps were generated to visualize the DEGs (Figure 1B-C). Both datasets were plotted using Venn diagrams and 24 ferroptosis-related DEGs were selected (Figure 1D). The Log2-fold change value and adjusted *P*-value of these 24 genes were also shown in **Table 3.** Figure 1E displayed the heatmap of these 24 ferroptosis-related DEGs based on the TCGA datasets We then sequenced 24 ferroptosis-related DEGs with varying degrees of correlation (Figure 1F) and conducted GO and KEGG enrichment analyses to examine the functions of the selected genes. The study found these 24 DEGs were mainly enriched in processes related to homocysteine and sulfur amino acid metabolism, cellular response to oxidative stress, and the Vitamin D receptor pathway (Figure 1G-J). Subsequently, an interaction network of these 24 DEGs was established using the STRING database (Figure 1K). Furthermore, the overall correlations of the 24 DEGs were also analyzed (Figure 1L).

### Developing and assessing the novel three gene risk-score signature

**Figure 2:**
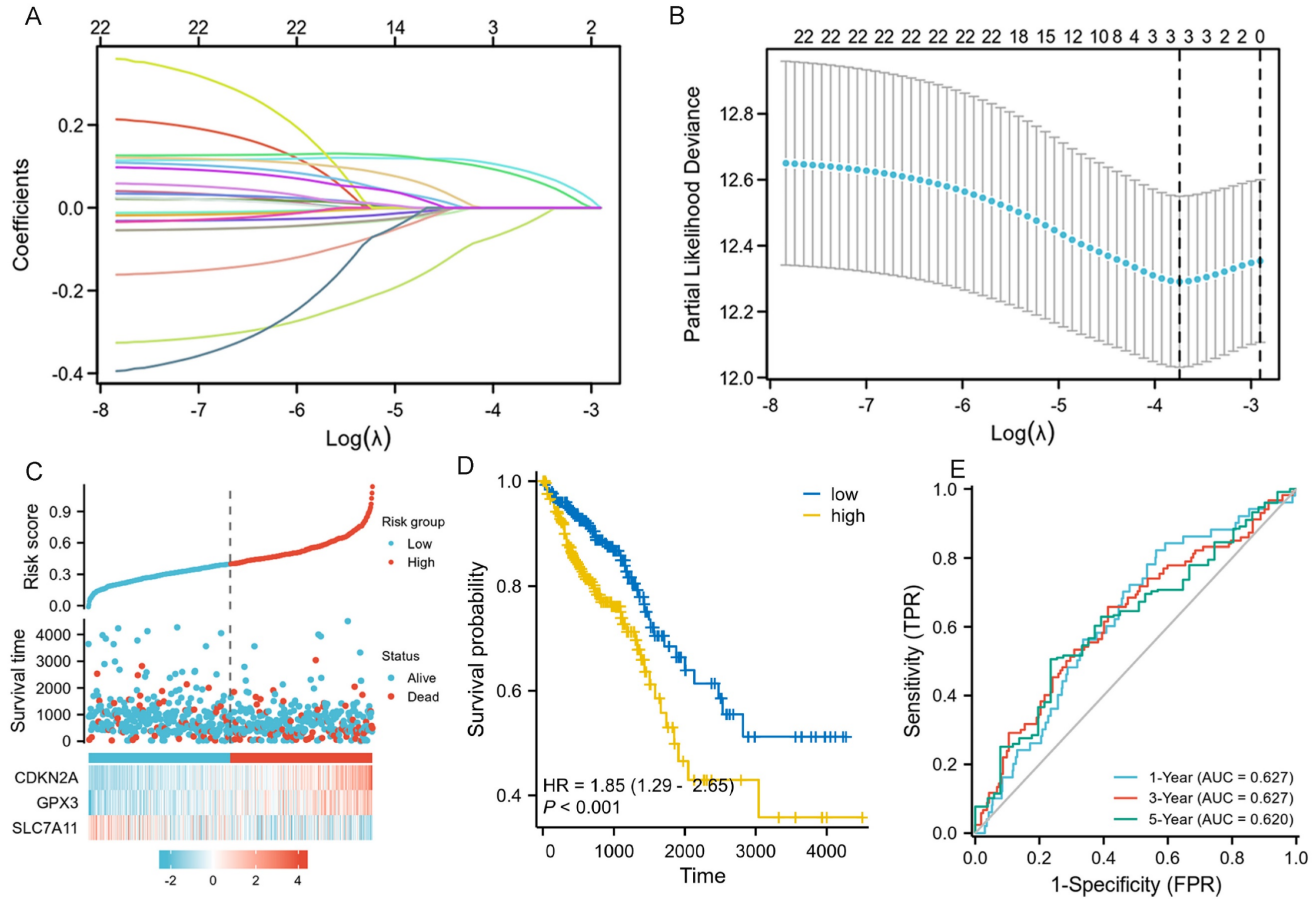
LASSO regression and risk score calculation. A: Coefficient value of 24 ferroptosis-related DEGs. B: Partial likelihood deviance of 24 ferroptosis-related DEGs. C: Risk score and survival time distributions, and heatmaps of gene-expression levels of the ferroptosis-related signature based on the TCGA data. D: Kaplan-Meier analysis suggests the survival outcome in the high-risk and low-risk groups in the TCGA cohort. E: ROC curves and AUC values of the risk score model for predicting the 1-, 3-, and 5-year OS times in the TCGA cohort.

We performed LASSO, COX regression analysis on these 24 ferroptosis-related DEGs. Finally, a total of three ferroptosis-related genes were identified. The formula for calculating the risk score: Risk score= (0.0853 × CDKN2A expression level) + (0.0745 × GPX3 expression level) + (-0.0413 × SLC7A11 expression level). The gene set was optimized using regression coefficient calculations (Figure 2A-B). The survival curve distribution of the risk model was analyzed. The coefficients were determined through 10-fold cross-validation, and GPX3, CDKN2A, and SLC7A11were identified as the most predictive variables based on minimum criteria.

Patients categorized as high risk exhibited a statistically significant decrease in survival rate compared to those categorized as low risk (Figure 2C). Moreover, Kaplan-Meier analysis and log-rank testing revealed a significant reduction in OS time for high-risk patients compared to low-risk patients (HR=1.85, *P*<0.001, Figure 2D). In addition, the ROC curves of this signature exhibited AUC values of 0.627, 0.627, and 0.620 at 1, 3, and 5 years, respectively (Figure 2E). GSEA analysis was conducted to investigate the potential influence of selected 3 ferroptosis-related gene expression levels on the transcriptomic profiles of both high-risk and low-risk groups of CRC. The results showed that pathways related to Fatty Acid Metabolism, IL6_JAK_STAT3 signaling, IL2_STAT5 signaling, WNT_β-catenin Signaling, Apical Junction, myogenesis, KARS Signaling, and interferon-gamma response were enriched in the high-risk population (Supplementary figure 1).

### The validation of CDKN2A, GPX3 and SLC7A11 mRNA expression in patients with CRC

**Figure 3:**
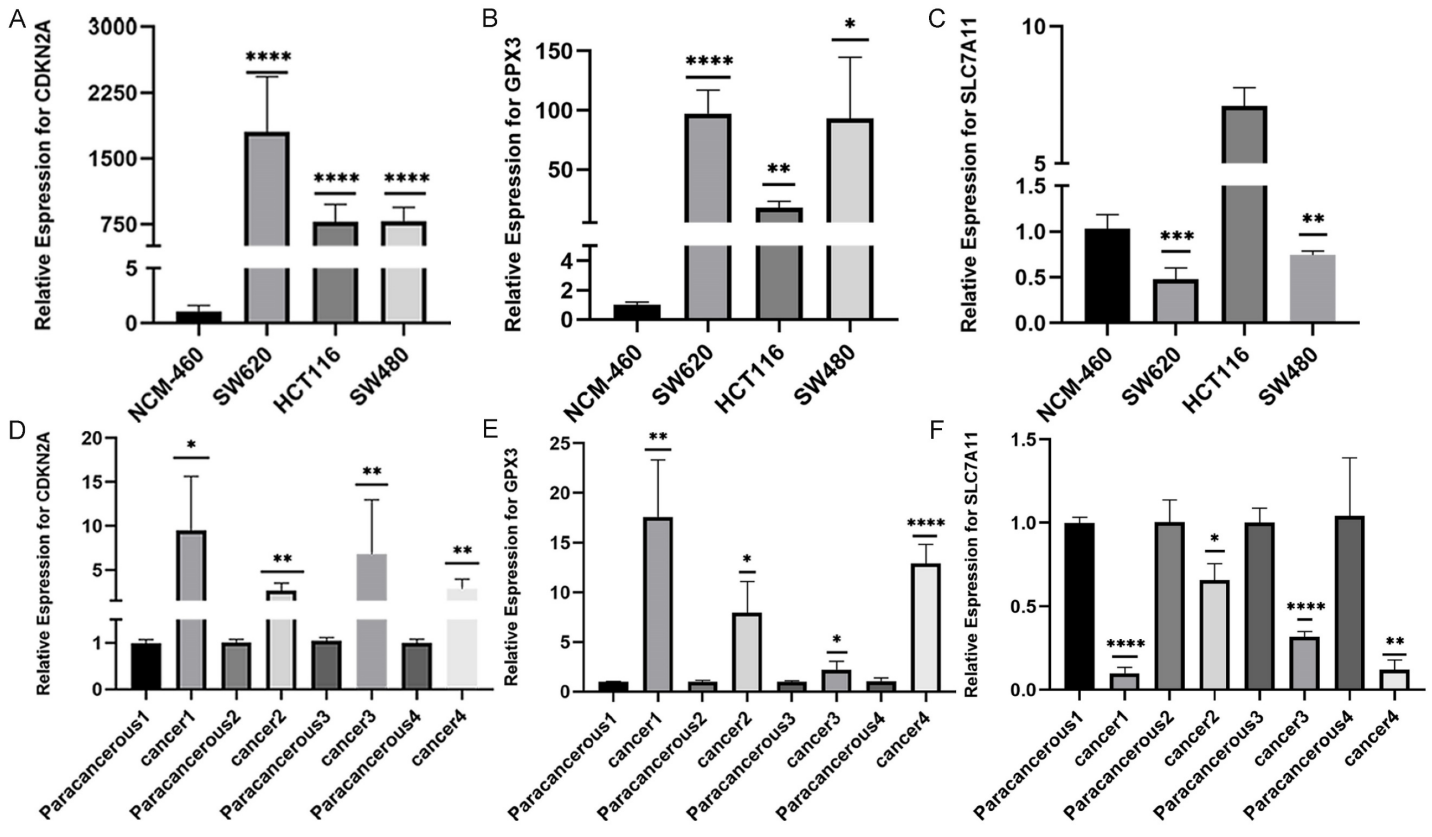
The validation of CDKN2A, GPX3 and SLC7A11 mRNA expression in patients with CRC. A-C: The validation of CDKN2A, GPX3 and SLC7A11 mRNA expression in several CRC and normal cell lines. D-E: The validation of CDKN2A, GPX3 and SLC7A11 mRNA expression in CRC and normal tissues. (Statistical significances were calculated using independent sample t-test. *P<0.05, **P<0.01, ***P<0.001, ****P<0.0001).

We conducted RT-qPCR analysis on three colorectal cancer cell lines (SW480, SW620, and HCT116) and one normal human colonic epithelial cell line (NCM460) to validate the aforementioned hypothesis. The results revealed that the observed mRNA expression of GPX3、CDKN2A and SLC7A11 in CRC cell lines was almost consistent with the aforementioned bioinformation results (Figures 3A-C). The expression of GPX3 and CDKN2A mRNA was significantly higher in CRC cell lines than in normal human colonic epithelial cell line. However, SLC7A11 had opposite results. These results were subsequently validated in tissues from patients with colorectal cancer. We collected 4 pairs CRC and normal tissues samples, and validated the mRNA expression of these three selected genes. As a result, we found that the expression of CDKN2A mRNA and GPX3 mRNA was significantly higher in CRC tissues than in normal tissues, the expression of SLC7A11 mRNA was significantly lower than in normal tissues (Figure 3D-F).

### The diagnostic values of the three selected ferroptosis-related genes and their relationships with tumor immunity

**Figure 4:**
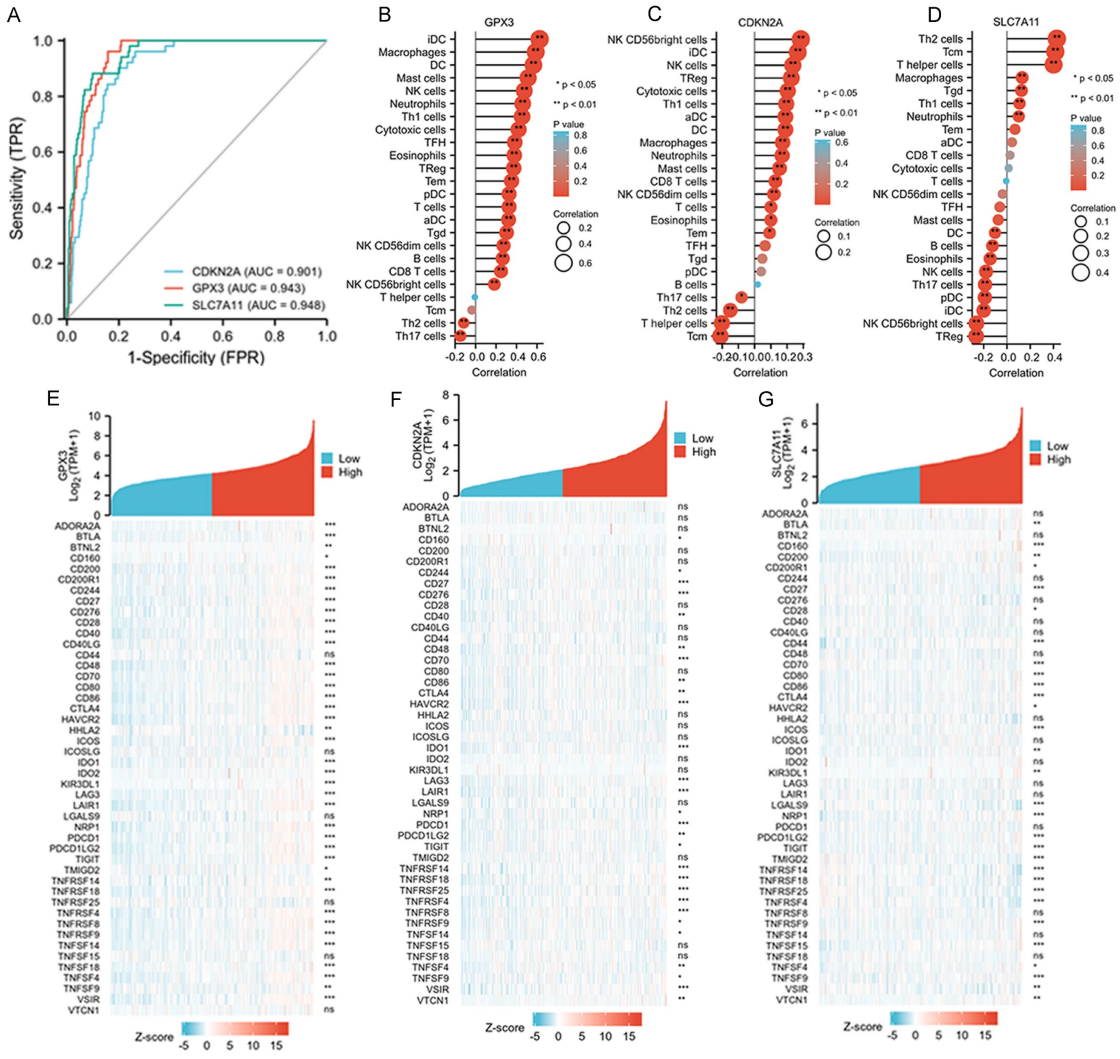
Associations between clinicopathologic features, tumor immunity and three selected ferroptosis-related genes in the TCGA-COAD dataset. A: ROC and AUC of CDKN2A, GPX3 and SLC7A11 to diagnose death for patients with CRC in the TCGA-COAD database. B-D: The relationships between GPX3, CDKN2A and SLC7A11 expression and infiltrating immune cells in the TCGA-COAD database. E-G: The relationships between GPX3, CDKN2A and SLC7A11 expression and CRC immune checkpoint genes of TCGA-COAD database.

The diagnostic values of GPX3, CDKN2A, and SLC7A11 for death were evaluated using ROC curve analysis, and we found all three genes exhibited satisfactory diagnostic potential for death (Figure 4A-D). Furthermore, we analyzed the correlations between the immune cell fraction of each sample, as precomputed in the single-molecule and cloud data, to compare the correlations of the three genes with immune cells. The five immune cells that exhibited the strongest correlation with these three genes are listed. GPX3 was more closely related to IDC, macrophages, DC, and mast cells; CDKN2A was more closely related to NK CD56bright cells, IDC, NK cells, TReg, Cytotoxic cells, and TH1 cells. SLC7A11 was found to be more correlated with TH2 cells, TCM, T helper cells, macrophages, and Tgd (Figure 4B-D). Finally, approximately 50 immune checkpoint-related genes were assessed in relation to GPX3, CDKN2A, and SLC7A11 to investigate their involvement in CRC (Figure 4E-G). Heat map analysis of single gene co-expression revealed a strong correlations between GPX3 and ADORA2A, BTLA, CD200, CD200R1, and CD244; CDKN2A have higher correlations with CD276, CD27, CD70, HAVCR2, IDO1; SLC7A11 have higher correlations with CD160, CD27, CD44, CD70, CD80.

### Spatial transcriptome analysis and single-cell localization of the three selected ferroptosis-related genes

**Figure 5:**
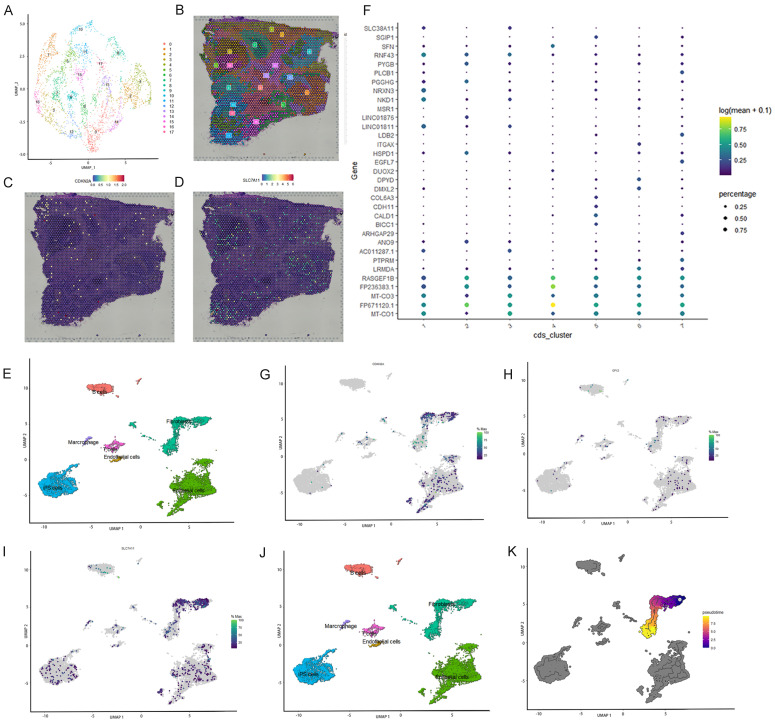
Single cell localization of three selected ferroptosis-related genes and spatial transcriptome validation. A-B: Seventeen clusters were identified by t-SNE and uniform manifold approximation and projection (UMAP). C-D: Feature plots and spatial feature plots were utilized to illustrate the distribution expression of CDKN2A and SLC7A11. E: Seven major cell clusters were identified via UMAP. F: Dot plot of cells proportion in the respective cluster expressing selected marker genes (dot size), and average expression (color scale). G-I: UMAP plot of GPX3, SLC7A11 and CDKN2A expression across all cell clusters. J: Pseudo-time trajectory and modules of genes whose expression varied with pseudo-time. K: Pseudo-time analysis was used to plot the early-stage trajectories of CRC.

Spatial transcriptome and single-cell sequencing analyses were used to evaluate the expression pattern and localization of GPX3, SLC7A11, and CDKN2A in CRC. The single-cell correlation technique enables the identification of cell types and tissue heterogeneity. Spatial transcriptomics enables the simultaneous acquisition of gene expression and spatial information, but not at the single-cell level. Integrating single-cell detection with spatial transcriptome detection enhances the precision of 3D state characterization, thereby expanding potential avenues for future investigations. First, spatial transcriptome analysis was conducted using a publicly available database. Seventeen clusters were identified using UMAP analysis (Figure 5A-B). Feature plots and spatial feature plots demonstrated the high expression of CDKN2A and SLC7A11 in CRC tissues. GPX3 was not found in the public database. The spatial expression and distribution of CDKN2A and SLC7A1 in CRC tissues were mapped using this database (Figure 5C, D).

Subsequently, single-cell sequencing was performed to determine the expression of these three genes across distinct cell types. Seven primary cell clusters were identified using UMAP analysis (Figure 5E). No clusters were exclusively derived from a single individual. An additional dot plot is shown in Figure 5F. The seven single-cell subcohorts were identified as “Epithelial cells,” “iPS cells,” “Fibroblasts,” “B cells,” “T cells,” “Marcrophage,” and “Endothelial cells,” in sequential order. The results indicated that SLC7A11 was predominantly enriched in subpopulations of Fibroblasts, Epithelial cells, and iPS cells. CDKN2A was predominantly enriched in subpopulations of Fibroblasts and Epithelial cells. However, the distribution of GPX3 expression was diffuse, and the level of expression was low. Furthermore, we evaluated the density profiles of the three genes using single-cell sequencing (Figure 5G-I). Pseudotime analysis was used to depict gene expression patterns during the initial stages of CRC development (Figure 5J). Based on pseudo-temporal data, we investigated the correlation between the expression of the three genes and the early stage of CRC, suggesting that the expression of SLC7A11 and CDKN2A may be significantly associated with the development and onset of early-stage CRC (Figure 5K).

### Construction and validation of nomogram for prognostic prediction

**Figure 6:**
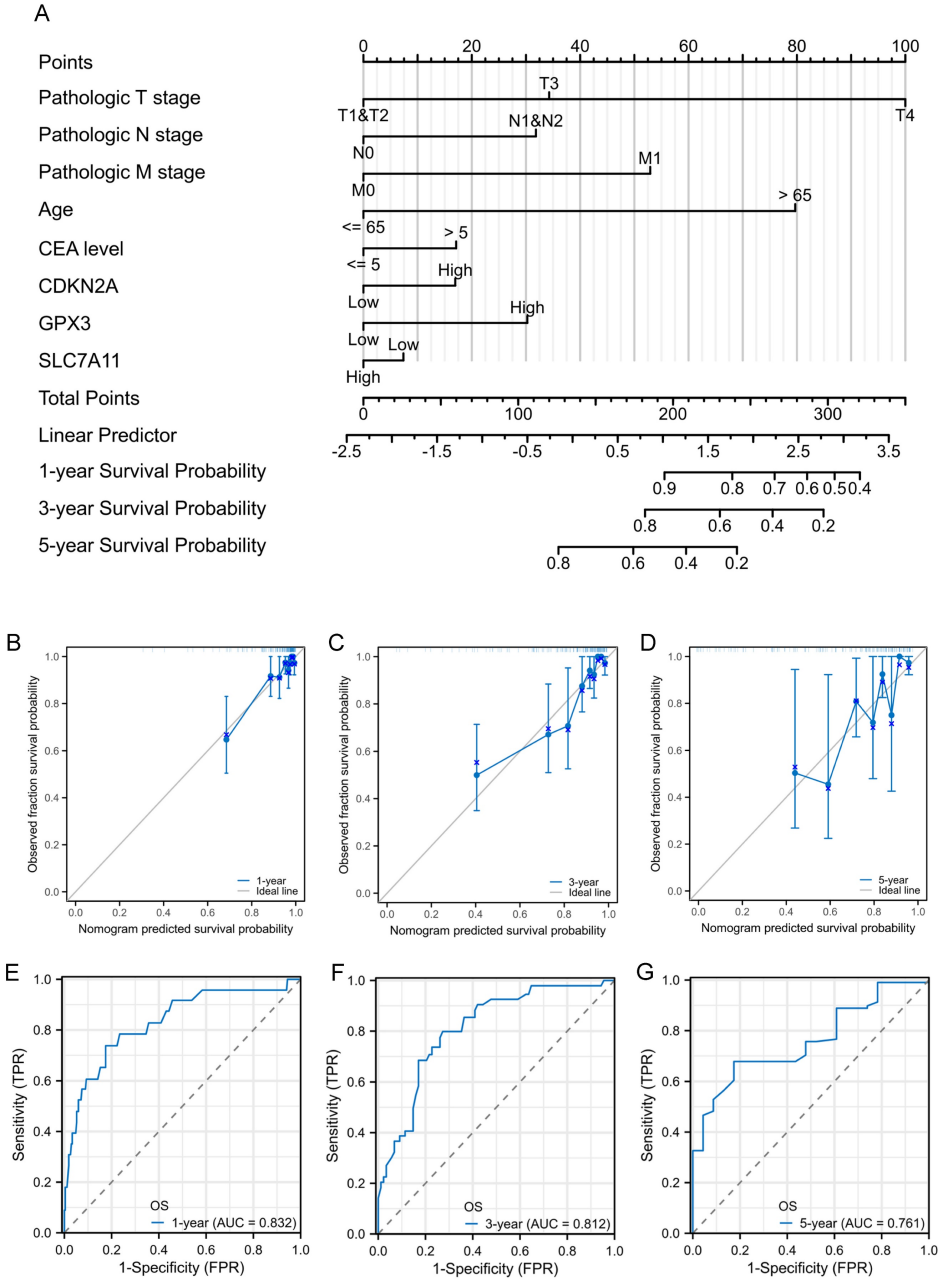
Prognostic nomogram for the 1-, 3-, and 5-year survival times of patients with CRC. A: Independent risk factors screened by multivariate Cox regression in the TCGA cohort were integrated into the nomogram model. B-D: Calibration curves of the nomogram for predicting 1-, 3-, and 5-year OS in the TCGA cohort. E-G: ROC curves and AUC values of the nomogram for predicting 1-, 3-, and 5-year OS.

To facilitate the clinical practice, we further converted the complex mathematical model into a nomogram. A nomogram was constructed to predict the 1-, 3-, and 5-year survival of patients with CRC using factors such as T stage, N stage, M stage, age, CEA level, CDKN2A expression, GPX3 expression, and SLC7A11 expression (Figure 6A). Calibration curves were created to predict the clinical outcomes for the 1-, 3-, and 5-year periods with reasonable accuracy (Figure 6B-D). The predictions made by the model were close to the actual outcomes. Subsequently, the ROC curves and area under the curve (AUC) values of the 1-, 3-, and 5-year OS were predicted (Figure 6E-G). The ROC curves are significantly above the diagonal dashed lines, with AUC values above 0.7. The above results showed that this nomogram had significant high prognostic prediction ability for the survival outcomes of patients with CRC.

### Abnormal DNA methylation of ferroptosis-related genes and miRNA-mRNA -TFs networks of ferroptosis in CRC

By the way, we analyzed the DNA methylation levels of *GPX3*, *CDKN2A*, and *SLC7A11* in CRC. DNA methylation heatmaps for the CpG sites in these three genes were also plotted. The CpG sites of *CDKN2A* (cg18849169, cg26891370, cg04201367, cg11109721, and cg22005145), *GPX3* (cg12840719 and cg04026675), and *SLC7A11* (cg06623625, cg02734904, cg06206831, cg21877274, cg01309945, cg04474257, and cg24869834) exhibited elevated methylation levels in CRC (Supplementary figure 2A-C). Methylation analysis of the *GPX3*, *CDKN2A*, and *SLC7A11* promoter regions, along with the corresponding expression data, was further conducted using publicly available datasets from the UALCAN database. The results revealed that the methylation levels of *CDKN2A* and *GPX3* promoters were significantly higher in CRC tissues than in normal tissues. In contrast, *SLC7A11* exhibited the opposite trend (Supplementary figure 2D-F). We lastly predicted miRNAs and TFs for three genes using TargetScan Human 7.2 and GeneCards databases. Based on these 28 TFs and three miRNAs, the TFs-mRNA-miRNA network was constructed, as shown in Supplementary figure 2G. We may explore the deep regulation mechanism of the three selected ferroptosis-related genes in the development of CRC based on this result.

## Discussion

Ferroptosis is primarily induced by the abnormal metabolism of amino acids, lipids, or iron, resulting in the excessive accumulation of L-ROS. This phenomenon can occur in both normal and tumor cells. The intracellular levels of iron and GSH in cancerous cells compared to those in normal cells can be utilized for targeted therapy based on ferroptosis 35, 36. Ferroptosis has demonstrated potential in the development of CRC and even in CRC therapy 37-39. However, the actual regulation mechanisms are still not very clear. Therefore, identifying the novel driving genes of ferroptosis can significantly contribute to the exploration of CRC development and the clinical treatment of CRC. In this study, we collected gene expression and clinicopathological data from TCGA dataset. Initially, 24 DEGs related to ferroptosis were identified. A prognostic model was developed using LASSO and Cox regression analyses, which identified three genes, GPX3, CDKN2A, and SLC7A11 as novel potential prognostic biomarkers.

*CDKN2A* mainly encodes two proteins: p14 and p16. p16 functions as a tumor suppressor by inhibiting cell cycle progression from the G1 to the S phase, thereby slowing down cell division. p14, a splice variant of CDKN2A, is significantly associated with cancer poor prognosis 40. CDKN2A has multifaceted involvement in cancer, encompassing its regulatory function and effects on different cancer types. However, there are few studies which explore the role of ferroptosis-related CDKN2A in the progression of CRC. Specifically, Xing X *et al.* found that the level of methylation of *CDKN2A* gene was an effective predictor of poor survival outcomes of patients with CRC 41. Also, Shi WK *et al.* found that CDKN2A has a pro-tumor effect in CRC. In CRC cell lines, silence the expression of CDKN2A can significantly inhibit the proliferation, induce cell apoptosis, arrest cell cycle and affect the process of epithelial-mesenchymal transition 42. Furthermore, Dong Y *et al.* found that high expression of CDKN2A can not only contribute to a poor survival outcome of patients with CRC, but also may guide PD-1 mediated immunotherapy for patients with CRC 43. In short, our data validated a significant correlation between the contributing role of CDKN2A and the development of CRC. However, the specifical molecular mechanisms of CDKN2A in the malignant progression of CRC are still unclear and need further investigation. More validated experiments are required to be performed in the future.

GPX3 could catalyze the reduction of organic hydrogen peroxide and hydrogen peroxide (H_2_O_2_) through the action of glutathione. Research indicates that the dysfunction of GPX3 expression in tumor cells is linked to a poor prognosis for patients with cancer and chemotherapeutic resistance 44, including endometrial adenocarcinoma 45, lung cancer 46, gastric cancer 47, prostate cancer 48, cervical cancer 49, and thyroid cancer 50. Among these kinds of solid cancer, GPX3 can not only play a cancer-promoting role, but also play a cancer-suppressing effect. However, there few researches about the role of GPX3 in the development of CRC. Pelosof L *et al.* found that GPX3 promoter methylation may predict platinum sensitivity in patients with CRC 51. Haug U *et al.* has also found that genetic variability in GPX3 may contribute to risk of rectal cancer but not of colon cancer 52. In conclusion, our research is the first study to explore the expression and prognostic effect of GPX3 in patients with CRC. However, the specific function and molecular mechanism of GPX3 in the development of CRC still need to be further explored.

Many studies have shown that SLC7A11 is one of the key regulators of ferroptosis 11, 53-55. Its main biological role is to control the transmembrane exchange of cystine and glutamic acid, and to mediate the entry of cystine into cells, thus providing raw material for intracellular GSH synthesis. Then, GSH may convert lipid peroxidation into non-toxic fatty alcohols, which protect cells from oxidative stress 56, 57. The expression of SLC7A11 is dysregulated in many kinds of tumor cells, such as lung cancer, liver cancer, breast cancer and CRC, and plays an important role in the occurrence and development of tumor. Inhibition of SLCA11 expression can affect the growth and drug sensitivity of many kinds of tumors. However, in different condition, the effect of SLC7A11 on the development of cancers may be different 58.

Some researchers found that SLC7A11 was overexpressed in various types of tumors, and the silence of SLC7A11 expression may increase the level of intracellular reactive oxygen species (ROS) and induce death of tumor cells 59, 60. However, other researchers observed that inhibition of SLC7A11 expression may contribute to the growth and proliferation of tumor cells. They found that tumor cells having high level of SLC7A11 expression were more sensitive to the level of glucose and glutamine restrictions than these cells with low level of SLC7A11 expression, which may due to the high nutrient dependence of tumor cells. Meanwhile, inhibition of SLC7A11 expression may contribute to tumor cell's adaptation to a hypoxic tumor environment, as a result, facilitating tumor development 61. The down-regulated expression of SLC7A11 can also increase the expression of multidrug-resistant proteins which can result in drug resistance 62. Just like our study, we found that the overall expression of SLC7A11 was lower in CRC than in normal tissues. Low expression of SLC7A11 may contribute to a worse survival outcome for patients with CRC. CRC cells with SLC7A11 low expression may more adaptive to the hypoxic tumor microenvironment and have more malignant phenotypes. However, there were still other studies which found that the inhibition of SLC7A11 expression may promote ferroptosis of CRC cells, then inhibit the malignant development of CRC and even CRC stem cells 63-67. From what we have discussed above, we can conclude that the actual action mechanism of SLC7A11 in the malignant development of CRC may be very complex, we need perform more experiments to make it clear in the future.

In this study, a risk-score signature and a prognostic prediction nomogram were also constructed to accurately predict the 1-, 3-, and 5-year OS rates. The established prediction model based on Lasso-Cox regression still had large predictive advantages. To validate our results again, OS survival curves were plotted and found that patients with different risk had significantly different outcomes. Furthermore, the calibration plot of the nomogram showed favorable consistency between the prediction of the nomogram and actual observations. Also, the time-dependent AUC (>0.7) indicated satisfactory discriminative ability of the nomogram. Just like we have stated, ferroptosis is a prominent subject in tumor research. However, the regulatory mechanisms linking tumor immunity and ferroptosis remain unclear. In our study, KEGG and GO analyses of DEGs in high-risk and low-risk groups reveal immune-related biological pathways and functions. High-risk patients demonstrate increased expression of immune responses and tumor progression pathways. A potential correlation between ferroptosis and tumor immune function can be deduced. Immunological checkpoint analysis revealed a positive correlation between risk score and immune checkpoint protein expression levels. To further investigate the expression of ferroptosis-related genes, we used spatial transcriptome validation and single-cell localization to analyze the expression patterns of the three genes in CRC. Our findings indicate that *CDKN2A* and *SLC7A11* are primarily expressed in fibroblast subpopulations. Pseudotime analysis predicts that the CDKN2A and SLC7A11 are closely related to the early stage of CRC. Collectively, we proposed that these three ferroptosis-related genes have significant implications for the development, prognosis, and clinical relevance of CRC, which provides a promising avenue for investigating the relationship between ferroptosis and CRC.

However, this study has some limitations. First, the primary focus of this study was the predictive methodology. The relevant gene expression in CRC cell lines and clinical specimens has been validated, but the related functional analysis requires further *in vitro* and *in vivo* assays. Second, further analysis is required to explore the conclusions drawn from single-cell and spatial transcriptome analyses. Third, further investigation is required to explore the action mechanisms of *GPX3*, *CDKN2A*, and *SLC7A11* in the development of CRC, including their upstream transcription factors and interacting proteins.

## Supplementary Material

Supplementary figures.

## Figures and Tables

**Table 1:** The sequences of primers.

Gene name	Sequences	
GPX3	Forward	5'-CAAGAGAAGTCGAAG ATGGACTG-3'
	Reverse	5'-GGGGATGTACTCCTCCCCAT-3'
CDKN2A	Forward	5'-GGGGTCGGGTAGAGGAGG-3'
	Reverse	5'- GCCCATCATCATGACCTGGA-3'
SLC7A11	Forward	5'-GGAGAAGGAATTCCAGGTCAT-3'
	Reverse	5'- AGCAATACAAGGAAGCCTTAGGT-3'
β-actin	Forward	5'- GTGGACATCCGCAAAGAC-3'
	Reverse	5'- AAAGGGTGTAACGCAACTA-3'

**Table 2:** Clinical characteristics of patients with CRC from TCGA-COAD dataset.

Characteristic	Levels	Overall
n		619
T stage, n (%)	T1	20 (3.2%)
	T2	105 (17%)
	T3	422 (68.4%)
	T4	70 (11.3%)
N stage, n (%)	N0	351 (57%)
	N1	150 (24.4%)
	N2	115 (18.7%)
M stage, n (%)	M0	459 (84.1%)
	M1	87 (15.9%)
Pathologic stage, n (%)	Stage I	105 (17.5%)
	Stage II	227 (37.9%)
	Stage III	179 (29.9%)
	Stage IV	88 (14.7%)
Gender, n (%)	Female	289 (46.7%)
	Male	330 (53.3%)
Race, n (%)	Asian	12 (3.3%)
	Black or African American	65 (17.6%)
	White	292 (79.1%)
Age, n (%)	≤65	269 (43.5%)
	>65	350 (56.5%)
BMI, n (%)	<25	98 (32.2%)
	>=25	206 (67.8%)
Residual tumor, n (%)	R0	450 (91.5%)
	R1	6 (1.2%)
	R2	36 (7.3%)
CEA level, n (%)	≤5	252 (63.5%)
	>5	145 (36.5%)
Perineural invasion, n (%)	No	172 (74.1%)
	Yes	60 (25.9%)
Lymphatic invasion, n (%)	No	331 (59.3%)
	Yes	227 (40.7%)
History of colon polyps, n (%)	No	364 (68.4%)
	Yes	168 (31.6%)
Colon polyps present, n (%)	No	207 (69.5%)
	Yes	91 (30.5%)
Neoplasm type, n (%)	Colon adenocarcinoma	454 (73.3%)
	Rectum adenocarcinoma	165 (26.7%)
OS event, n (%)	Alive	492 (79.5%)
	Dead	127 (20.5%)

**Table 3:** The selected 24 ferroptosis-related DEGs.

Gene name	Log2-fold change value	Adjusted *P*-value
ALB	2.770574661	1.07E-58
CA9	2.308236488	4.31E-47
CBS	2.109997134	2.35E-06
CDKN2A	2.217311987	3.65E-15
CP	2.238971828	9.95E-21
DPEP1	-2.33747343	4.18E-15
ETV4	-2.41166948	2.06E-30
GDPD5	4.140235566	2.76E-25
GPX3	3.880235305	5.23E-46
HCAR1	3.682487512	5.68E-63
HILPDA	2.066355368	5.35E-39
KRT16	5.849761786	3.60E-72
KRT6B	5.060435957	1.89E-65
LCN2	5.121558867	2.23E-10
MT1G	5.725881403	3.80E-36
MYCN	6.123645098	5.03E-58
NGB	2.543142061	8.06E-79
NOX4	2.826449302	4.88E-53
SCD	-3.29715582	1.97E-41
SLC7A11	-2.0894971	7.07E-18
TFAP2A	-3.63533677	4.23E-42
TUBB4A	4.082463138	6.25E-17
TYRO3	-5.58165687	9.02E-19
UCHL1	3.548301809	1.31E-35
