# Supplementary material for: Construction and Validation of Novel Ferroptosis-related Risk Score Signature and Prognostic Prediction Nomogram for Patients with Colorectal Cancer
Source: Int J Med Sci. 2024 Apr 22;21(6):1103–16. doi: 10.7150/ijms.91446 (PMC11103399; doi:10.7150/ijms.91446)

Supplementary figure 1 Single-gene GSEA analysis of these three selected genes.

A-C: Single-gene GSEA analysis of CDKN2A

D-F: Single-gene GSEA analysis of GPX3

G-I: Single-gene GSEA analysis of SLC7A11

Supplementary figure 2. Heat map of original DNA methylation measurements at CpG sites in three genes and miRNA-mRNA-TFs interaction networks of the three selected ferroptosis-related genes.

A-C: The heat map of DNA methylation at CpG sites in the CDKN2A, GPX3 and SLC7A11 gene by the MethSurv database.

D-F: Promoter methylation level of CDKN2A, GPX3 and SLC7A11 in normal tissues and primary CRC tissues analyzed by the UALCAN database.

G: TFs-mRNA-miRNA networks of the ferroptosis genes (28TFs、3 mRNAs and 3 miRNAs were included).

Supplementary figure 1

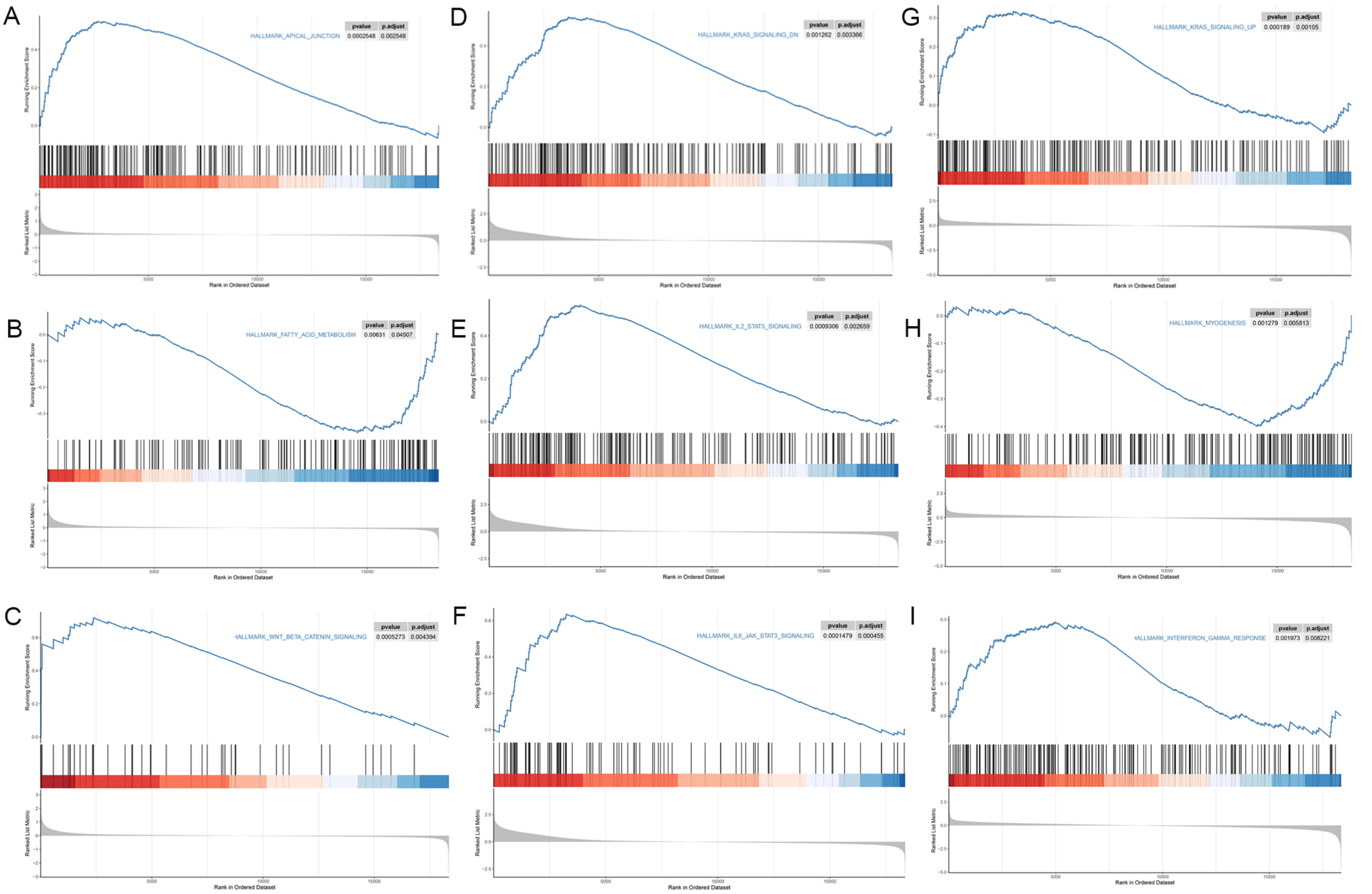

Supplementary figure 2

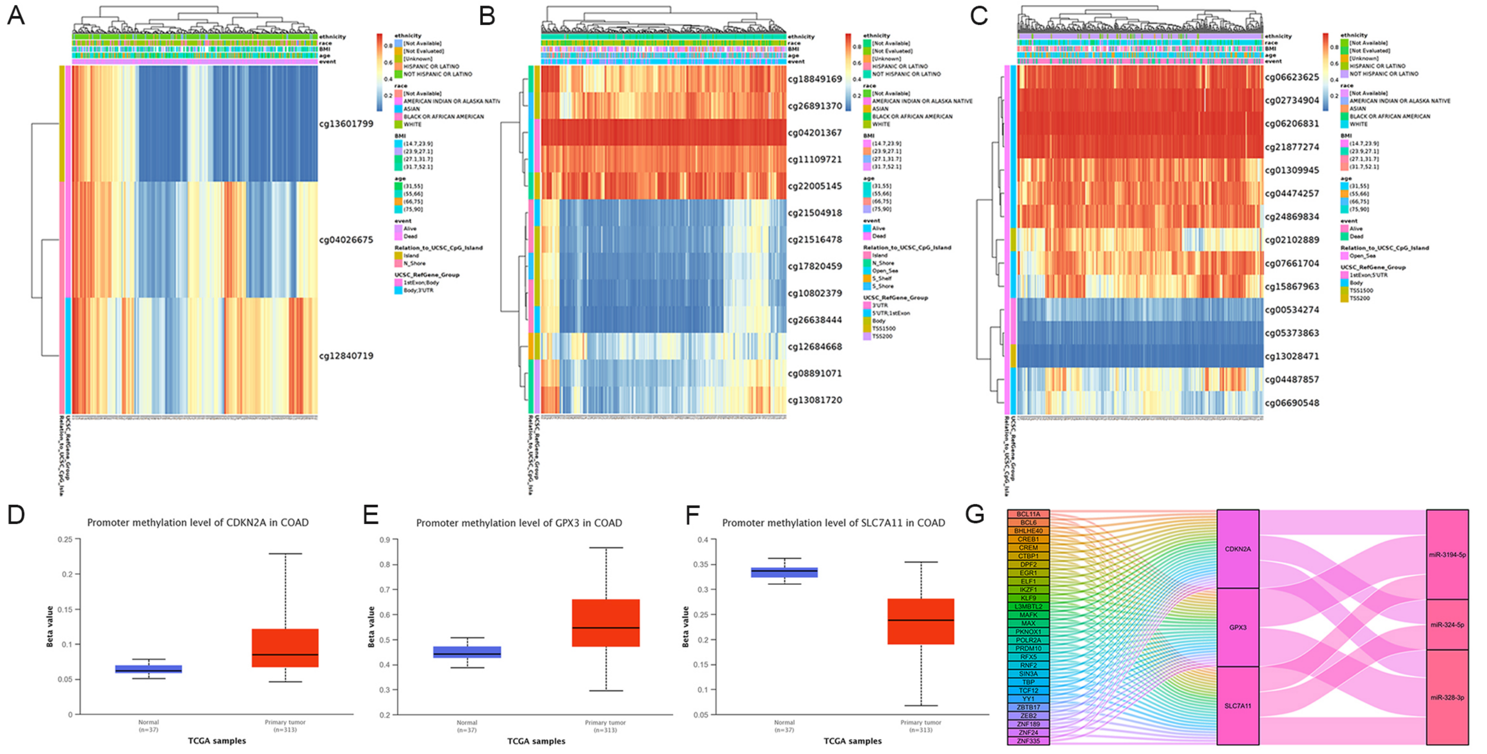

Supplement: Supplementary file 1 — Supplementary figures. [file ijmsv21p1103s1.pdf]
